# The Inhibition and Equilibrium of Policy Effectiveness of the Low-Carbon Economy: Evidence from Liaoning Province of China

**DOI:** 10.3390/ijerph20053961

**Published:** 2023-02-23

**Authors:** Rongxin Chen, Xinyuan Shi

**Affiliations:** School of Law, Xiamen University, Xiamen 361005, China

**Keywords:** low-carbon economy, policy, multi-factor linkage mechanism, Chinese experience

## Abstract

Excessive emissions of carbon dioxide and other greenhouse gases have seriously affected the ecological environment, public health, and the normal operation of the social economy, and the development of the low-carbon economy has become an international consensus. The policy norms are one of the important factors affecting the development of the low-carbon economy; however, the implementation of low-carbon economic policy in many countries has been inhibited. This study chose Liaoning Province of China for the case study, and the policy system, the policy tools, the administrative system, the low-carbon technology, and the low-carbon concept are found to be factors that led to the inhibition of the policy effectiveness of the low-carbon economy in Liaoning Province. We applied the modified Schweller Neoclassical Realist Theory to establish a multi-factor linkage model to demonstrate the overall relationship among various variables. The results show that the equilibrium of policy effectiveness of the low-carbon economy in Liaoning Province depends on different permutations of variables. We also discussed the problems of the policy system, the policy tools, the administrative system, the low-carbon technology, and the low-carbon concept that lead to policy effectiveness inhibition, and used the economic method to set a special mathematical model for maximizing the equilibrium of policy effectiveness of the low-carbon economy in Liaoning Province. In response to the problems of the above factors, strategies to promote the development of the low-carbon economy in Liaoning Province are proposed. This study enriches the research on the policy effectiveness of the low-carbon economy in China and provides some inspiration for the goal of carbon neutrality and other developing countries with high carbon emissions.

## 1. Introduction

The low-carbon economy, which was first proposed by the UK government in its Energy White Paper (2003) “Our Energy Future—Creating the Low-carbon Economy”, means that the low-carbon economy aims to create economic output by adjusting the energy mix and improving energy efficiency [1]. Some literature finds that low-carbon economic policy can have a positive impact on public health. For example, well-designed initiatives that curb greenhouse gas emissions in energy, residential construction, urban transport, and agricultural systems can enhance global public health [2].

Developing a low-carbon economy is an important goal for China. China is the largest emitter of carbon dioxide gas in the world, with 10,668 million metric tons emitted in 2020 [3], making the development of the low-carbon economy an important task for China in 2012. In that year, the 18th National Congress of the Communist Party of China proposed to “vigorously develop a low-carbon economy and improve related policies”. Among these, the implementation of low-carbon economic policy in Liaoning Province has attracted much attention and has achieved a certain landmark status nationwide. It should be noted that Liaoning Province, which is located in the northeast of China, has received much attention for its low-carbon economic policy because Liaoning Province has more severe problems of resource scarcity, shrinkage, and depletion compared to other provinces in China. This is mainly because Liaoning Province relies more on coal for energy consumption, resulting in high CO_2_ emissions. In addition, Liaoning’s economic growth relies too much on heavy chemical industries, while the development of low-energy tertiary industries and high-tech industries lags behind, leading to high energy consumption in Liaoning Province [4]. Therefore, it is of great strategic and practical significance for Liaoning Province to develop a low-carbon economy to transform its economic development mode. Based on these reasons, this study selects Liaoning Province as the target case and carries out an in-depth analysis.

The Liaoning Provincial Government has proposed many low-carbon economy policies in recent years; however, the effectiveness of these policies has been inhibited in the following ways [5]:

(1) The energy consumption structure in Liaoning Province is still dominated by coal and oil. First, the proportion of coal consumption remains high in the energy consumption structure of Liaoning Province. Coal consumption accounted for 57.9% in 2019, which is 10% lower than in 2010, but the decrease in the proportion is on a decreasing trend; second, oil accounted for 31.7% in 2019, which is 4.7% higher than in 2010. Summing them up, the share of both coal and oil reached 89.6% in 2019, which is 6.1% higher than the national average. Third, other energy sources such as hydropower and nuclear power accounted for only 3.3% in 2019, which is 4.5% lower than the national average.

(2) The industrial structure in Liaoning Province is still dominated by highly energy-consuming industries. For a long time, the output value of heavy industry in Liaoning Province has accounted for more than 80% of the total industrial output value, while the energy consumption of heavy industry is huge. In particular, the industrial output value of the six high energy-consuming industries (According to the 2010 China National Economic and Social Development Statistics Report, the six major energy-consuming industries are as follows: chemical raw materials and chemical products manufacturing, ferrous metal smelting and rolling processing industry, non-ferrous metal smelting and rolling processing industry, non-metallic mineral products industry, petroleum coking and nuclear fuel processing industry, electricity production and supply industry.) showed an increasing trend among the total industrial output value, reaching 52.4% in 2019, an increase of 12.5% over 2015, while the energy consumption of the six high energy-consuming industries reached 89.6% in 2019.

The impact of the implementation of the Liaoning Provincial Government’s low-carbon economic development policy on industrial structure, energy structure, and emission reduction has attracted widespread attention at home and abroad. First of all, the Liaoning Provincial Government attached importance to the performance evaluation of low-carbon fiscal policy and established a financial performance evaluation system. According to the phased objectives of low-carbon economic development in Liaoning Province, the timeliness of various financial policy tools is reviewed regularly, and the effectiveness of the policies is evaluated according to the corresponding policy effectiveness, so as to adjust the direction and strength of the policies in a timely manner and produce the optimal policy effectiveness [6]. Secondly, the Liaoning Provincial Government has promoted emerging industries from the aspects of industrial planning, finance, and financial support, and some emerging industries have developed rapidly, especially in terms of fiscal and tax policies. Because Liaoning Province needs to treat low-carbon industry as a key industry and vigorously develop a low-carbon economy, it needs to expand the special funds for energy conservation in the financial budget [7]. Finally, the Liaoning Provincial Government will make a demonstration in the coastal cities of Liaoning Province, create a low-carbon economy pilot community, and implement the green community policy [8]. However, in general, most studies have only analyzed one of the factors that affect the policy effectiveness of the low-carbon economy in Liaoning Province. For example, Zhou and Zhou pointed out that the policy effectiveness of China’s low-carbon economy is closely affected by policy tools and believed that market economic tools are valuable in promoting the transformation of low carbon economy in pilot areas, while mandatory tools and voluntary tools failed to achieve the expected objectives [9]. Li et al. believed that the reasonable design of tax tools could promote the effectiveness of policies, and further suggested that the government should actively implement preferential tax policies or innovation subsidy policies for enterprises engaged in scientific research and innovation to ensure or promote the motivation of enterprises to carry out technological innovation [10]. Therefore, we must formulate scientific industrial development plans and effective fiscal and tax policies, vigorously develop low-carbon industries, and carry out low-carbon technology development and promotion and application, so as to promote the rapid development of Liaoning’s low-carbon economy.

In fact, there are many documents studying the government’s low-carbon economic policies, but these documents are more qualitative analysis than quantitative analysis. They fail to study the inhibition and equilibrium of the efficiency of low-carbon economic policies in depth and also fail to analyze the relationship between factors affecting the efficiency of policies. However, the effectiveness of low-carbon economic policy is not only subject to many factors but also may cause inhibition due to the irrational combination of factors, which will lead to more economic risks and environmental hazards. In this regard, it is necessary for this study to take Liaoning Province of China as the research target, creatively analyze the efficiency of the government’s low-carbon economic policy with the help of the modified Schweller’s Neoclassical Realism Theory, and use the multi-factor linkage mechanism model to show the combined efficiency of the antecedent variables, independent variables, and dependent variables, so as to more accurately grasp the inhibition and equilibrium law of the efficiency of low-carbon economic policy. Therefore, this study can make up for the shortcomings of existing research, and also has a certain theoretical innovation. The research results can provide an important reference and inspiration for the low-carbon economic decision-making of other provinces of China and even relevant countries.

The remainder of this paper is organized as follows. The qualitative analysis of the multi-factor linkage model and its theoretical underpinnings belongs to the coming section, followed by the discussion of the causes of the inhibition of policy effectiveness. The next segment outlines a special mathematical model that could maximize the equilibrium of the policy effectiveness of the low-carbon economy in Liaoning Province. The recommendation section is the last, where we propose specific countermeasures to maximize the equilibrium of policy effectiveness. On the one hand, from the perspective of research purposes, this study aims to construct a multi-factor linkage mechanism for the efficiency of the Liaoning Provincial Government’s policies for developing a low-carbon economy, looking for the potential relationship between the policy system, administrative system, policy tools, low-carbon technology, and low-carbon concept, so as to master how to influence the results of policy effectiveness through different combinations of variables. On the other hand, from the perspective of research value, through this multi-factor linkage mechanism, we can better reduce the degree of inhibition of low-carbon economic policy effectiveness, effectively improve the balanced level of policy effectiveness, and then provide decision-making suggestions for local governments to develop a low-carbon economy. In addition, some developing countries similar to China are also experiencing the dilemma of low-carbon economic policy, so this study can also provide better enlightenment and suggestions for these countries to introduce reasonable low-carbon economic policies. Of course, this study also has certain limitations and assumptions, and we will explain them accordingly.

## 2. Methodology and Variables

### 2.1. The Multi-Factor Linkage Mechanism

In this section, we establish a multi-factor linkage mechanism that demonstrates the overall relationship among various variables through the Modified Neoclassical Realist Theory.

Neoclassical Realist Theory, originally a methodological approach in the field of international relations and foreign policy studies, argues that various independent variables such as economic conditions, political systems, military power, and natural endowments can have an impact on foreign policy effectiveness [11]. Neoclassical Realist Theory helps governments choose the best foreign policy by establishing a multidimensional and comprehensive logical model that includes basic assumptions, empirical premises, and causal derivations [12]. It should be noted that there are many schools of Neoclassical Realist Theory, while the variable selection approach proposed by Schweller is applied to this paper. Theoretically, Schweller chose four main independent variables that may influence policy effectiveness: elite consensus, elite cohesion, social cohesion, and social consensus. Elite consensus and elite cohesion are internal independent variables that jointly influence the state’s willingness and implementation intention of policy formulation, while social cohesion and social consensus are external independent variables that jointly influence the ability and social conditions of policy implementation. Different combinations of these four independent variables determine the degree of internal coherence, which then leads to a basic hypothesis: the degree of coherence determines whether it can respond to the external pressure in a timely manner [13]. In other words, the degree of consistency affects policy choices. In summary, Schweller’s Neoclassical Realist Theory emphasizes the analysis of the relationship between different variables and how the combination of variables affects policy effectiveness. This methodology has important implications for this paper in establishing a multi-factor linkage mechanism to analyze the inhibition and equilibrium of policy effectiveness of low-carbon policy in Liaoning Province.

However, Schweller’s Neoclassical Realist Theory cannot be directly replicated and applied to this study for two reasons: first, the dependent variable mentioned by Schweller is different from the dependent variable of this study. The former’s dependent variable is foreign policy effectiveness, while the dependent variable in this study is the policy effectiveness of the low-carbon economy in Liaoning Province, which then determines that the independent variables are also fundamentally different. Second, Schweller’s Neoclassical Realist Theory focuses more on the influence of internal factors on policy choice, while ignoring the role of external factors on internal factors. On the contrary, this study additionally considers the influence of external factors on internal factors, making the multi-factor linkage mechanism more complete and rigorous. Therefore, this paper makes appropriate modifications based on Schweller’s Neoclassical Realist Theory.

### 2.2. Variables

Based on the modified Schweller’s Neoclassical Realist Theory, this paper chooses the low-carbon economic policy of Liaoning Province as the antecedent variable, the policy system and the policy tools as the external independent variables, the administrative system, the low-carbon technologies, and the low-carbon concept as internal independent variables, and the equilibrium degree of policy effectiveness as the outcome-dependent variable, so as to establish a new theory of multi-factor linkage mechanism.

#### 2.2.1. The Antecedent Variables

The low-carbon economic policy of Liaoning Province is the root of theoretical support and the starting point of logical derivation and thus is considered the antecedent variable.

#### 2.2.2. The External Independent Variables

The policy system and policy tools are external independent variables, which change with the change in the antecedent variables and are easily affected by the low-carbon economic policies of Liaoning Province. These external independent variables involve a lot of relevant departments, which are easy to change in a short time and have a certain fixity and independence. On the one hand, with regard to the policy system, systematic and targeted policies affect the effectiveness of policies [14]. The policy system refers to the sum of all low-carbon economic policies, such as economic policies, industrial policies, energy policies, technical policies, and social policies, which have their own priorities and complementarities between each other. Targeted policies require the Liaoning Provincial Government to formulate targeted and specific policies according to the development of the low-carbon economy in Liaoning Province. Because the development of a low-carbon economy is a public issue, the policy system needs to be constructed in a reasonable combination for the entire society. The policy system often involves many functional departments, including resources, energy, finance, environmental protection, and other departments. These departments urgently need to form a unified and complementary policy, otherwise, it may affect the effectiveness of low-carbon economic policies. From the perspective of horizontal structure, the policy system is divided into different types of subsystems, which complement, cooperate, and coordinate with each other, so that the policy system can maintain its own organic integrity. From the perspective of vertical structure, the policy system is divided into several levels from top to bottom. High-level policies are the basis of low-level policies, and low-level policies are the embodiment of high-level policies. Each constituent element of the low-carbon economy policy system has different regulatory objects and functions, and there is a complementary relationship between them. Therefore, they should coordinate and cooperate with each other in actual operation, so as to promote the coordinated development of the low-carbon economy.

On the other hand, the policy tool is not only an independent variable used by the government to influence policy effectiveness but also a policy measure to achieve the goal of low-carbon economic development. Policy instruments can be divided into three categories, namely fiscal instruments, tax instruments, and credit instruments [15]. Financial tools are the means by which the government provides financial subsidies. Tax tools are the means for the government to implement tax incentives and tax constraints. Credit instruments are the means by which the government stimulates financial institutions to provide credit support to low-carbon enterprises and research institutions. The government needs to rationalize the combination of a series of policy tools to form a scientific and accurate policy design, so as to promote the effectiveness of low-carbon economic policies.

#### 2.2.3. The Internal Independent Variables

The administrative system, low-carbon technology, and low-carbon concept are internal independent variables, which are less influenced by the antecedent variables, but all fluctuate within the framework of the policy effectiveness of the low-carbon economic policy of Liaoning Province. Firstly, the administrative system means the evaluation system of local government performance. The central government’s criteria for evaluating the performance of local governments affect the intrinsic motivation of the Liaoning Provincial Government to develop the low-carbon economy. According to the assumption of “economic man” rationality, local governments will decide their own behavior strategies based on their own interests [16]. Therefore, the administrative system should also be set up in accordance with this assumption. Secondly, regarding low-carbon technology, strengthening low-carbon technology innovation is an important foundation for developing the low-carbon economy. The low-carbon technologies can change the way energy is used, and they are closely influenced by scientific research funds and scientific research personnel [17]. Lastly, regarding the low-carbon concept, whether enterprises and individuals uphold the low-carbon concept fundamentally affects the policy effectiveness of the low-carbon economy. The low-carbon concept of enterprises is mainly reflected in the theory of corporate social responsibility (CSR). CSR refers to the positive impact a company has on society and the environment in the production of its products or the provision of its services [18]. Social responsibility is achieved through the cooperation of stakeholders such as employees, customers, shareholders, and suppliers.

#### 2.2.4. The Dependent Variable

**Figure 1:**
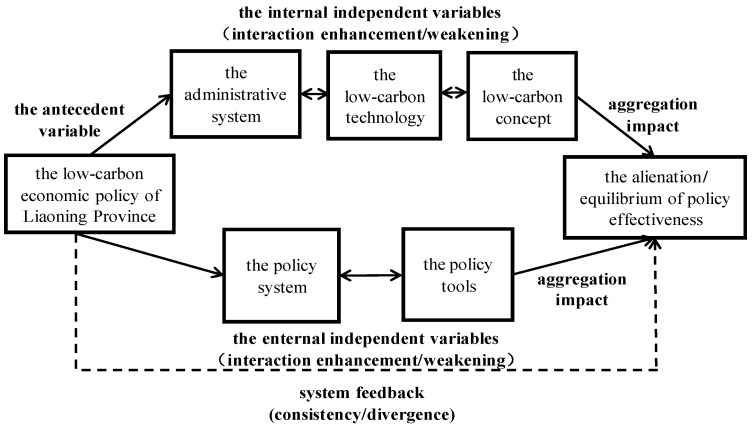
The multi-factor linkage mechanism of policy effectiveness of the low-carbon economy.

The equilibrium degree of policy effectiveness of the low-carbon economy in Liaoning Province serves as the dependent variable, which is affected by different permutations of the antecedent, external, and internal variables. For further detail on the multi-factor linkage mechanism of policy effectiveness of the low-carbon economy, we refer to Figure 1.

## 3. Causal Analysis

As mentioned above, the policy system, the administrative system, the policy tools, the low-carbon technologies, and the low-carbon concept are the factors that influence the policy effectiveness of the low-carbon economy in Liaoning Province, and thus we should explore the reasons for the inhibition of each factor.

### 3.1. The Policy System

The low-carbon economy policies in Liaoning Province are fragmented and confusing, and there is a lack of a general policy specifically for the low-carbon economy in Liaoning Province to serve as a guide. During the period 2013–2022, Liaoning Provincial Government enacted a series of policies such as the Liaoning Province Environmental Protection Regulations, the Liaoning Province 14th Five-Year Ecological Protection Plan, and the Liaoning Province Implementation Plan for Cleaner Production Audit (2021–2023). However, most of these policies are similar in terms of their content and provide for abstract but not specific rules, such as “accelerating the transformation and upgrading of traditional industries” [19]. In addition, these policies are almost isolated, which means they lack essential interaction with each other. So far, the Liaoning Provincial Government has not issued a special economic policy dedicated to promoting technological innovation [20], making it difficult to guarantee low-carbon technology innovation, which is a typical example of how economic policies and technology policies lack good coordination.

The policies are not targeted and specific and lack consideration of the current situation in Liaoning Province. In fact, the provisions of Liaoning Province’s low-carbon economy policies are basically copied from the national policies, without taking into account Liaoning Province’s situation, resulting in a lack of precision in policy implementation [21]. For example, the 14th Five-Year Ecological Protection Plan of Liaoning Province has a special section on “control of greenhouse gas emissions”, but the specific provisions are too abstract and not specific; they are basically the same as those in the National Ecological and Environmental Monitoring Plan (2020–2035).

### 3.2. The Policy Tools

Policy tools are the essential means to achieve policy goals. In general, the policy tools that affect the policy effectiveness of the low-carbon economy in Liaoning Province include fiscal tools, tax tools, and financial tools.

From the perspective of the financial tools, the state’s financial investment in the Liaoning Provincial Government is clearly insufficient. The energy structure of Liaoning Province has been dominated by coal and requires significant government investment to change this high energy-consuming tradition and improve the low-carbon technology innovation. Although the state has begun to pay attention to and invest in the development of the low-carbon economy in Liaoning Province, the state’s financial support for Liaoning Province is highly insufficient compared to other provinces. For example, in 2021, the state’s financial support to the Department of Ecology and Environment of Guangdong Province was RMB 406,450,800 [22], while the financial support to the Department of Ecology and Environment of Liaoning Province was only RMB 99,087,700 [23], which is equivalent to a quarter of the former. However, compared with Guangdong Province, it is more urgent for Liaoning Province, with more severe problems of resource scarcity, shrinkage, and depletion, to develop a low-carbon economy, and it deserves more financial support from the state.

Regarding taxation tools, firstly, the tax incentives in Liaoning Province are insufficient for the development of low-carbon industries. Some of the application conditions for tax incentives in Liaoning Province are unclear, resulting in the exclusion of many enterprises that should have enjoyed tax incentives. Taking the tax incentive measure of additional deductions for scientific research expenses as an example. In 2015, the top three provinces with the most total additional deductions for scientific research expenses were Guangdong Province, Jiangsu Province, and Zhejiang Province, with RMB 10.86 billion, RMB 5.96 billion, and RMB 5.15 billion, respectively, while the additional deduction for scientific research expenses in Liaoning Province was RMB 870 million, which is equivalent to only 0.9%, 14.6%, and 16.9% of the top three provinces, respectively [24]. The reason for this is that the Liaoning Provincial Government did not clearly define “scientific research activities”, which is the prerequisite for enjoying tax incentives. Considering the above factors, in practice, in order to ensure that the implementation of the policy does not make mistakes, the examination procedures of the taxation department are very cumbersome, such as requiring enterprises to submit many materials, resulting in the fact that enterprises are often reluctant to apply for tax incentives. Secondly, the punitive tax constraint is insufficient. The current taxation policy for excess energy consumption has the problems of unreasonable tax rate setting, narrow taxation scope, and few types of taxes. Specifically, on the one hand, the energy consumption structure of Liaoning Province is dominated by coal, but so far the state and Liaoning Province only levy the resource tax on coal but do not include coal consumption in the scope of consumption tax. Based on this, the problem is that the tax rate of the coal resource tax ranges from 2% to 10%, but the minimum tax rate of 2% is too low compared with the minimum tax rate of 6% for oil and natural gas, which can hardly play an effective role in limiting the consumption of coal [25]. On the other hand, there is a lack of independent taxes targeting carbon emissions. Although taxes such as the environmental protection tax, resource tax, and consumption tax can promote energy conservation and emission reduction, they are not directly designed for CO_2_ emission reduction, making it difficult to limit CO_2_ emissions. Setting up a carbon tax has already achieved considerable results abroad. For example, in 1991, Sweden set up a carbon tax as part of its energy tax reform, and then Sweden’s CO_2_ emissions were reduced by 15% in 1995, with 90% of the reduction in emissions associated with the imposition of the carbon tax [26].

Concerning financial tools, financial institutions do not provide enough credit support to low-carbon enterprises in Liaoning Province for several reasons: Firstly, the waiting period for the profitability of enterprises producing new energy-saving products is long [27], and the enterprises may end up with no profit or even a loss, which leads to the commercial banks and other institutions not daring to take the risk of approving loans for the low-carbon enterprises; secondly, the current financial policy in Liaoning Province does not provide for tracking and supervision of low-carbon credit, which leads to the financial institutions not being able to obtain sufficient information in a timely manner to find a quick solution to the problem once the enterprise is not operating well. Thirdly, there are few financial institutions in Liaoning Province, and the financial products are too simple, which leads to the situation that the capital scale of financial institutions in Liaoning Province is not enough to cope with the strong demand for loans from the low-carbon industries.

### 3.3. The Administrative System

The administrative system is the practical basis for the implementation of low-carbon economic policies. The administrative system is subject to many factors such as administrative concepts, administrative systems, administrative subjects, administrative capacity, and administrative measures. A perfect and feasible administrative system can maximize the resultant force of policies and better develop the effectiveness of low-carbon economic policies. Effective low-carbon economic policies need not only a good design but also the administrative power to implement policies. From the perspective of administrative concepts, in the past few years, the pursuit of GDP has been the cause of the inhibition of the effectiveness of Liaoning’s low-carbon economic policies. Gross domestic product (GDP) refers to the value of all final products and services produced by a regional economy in a quarter or a year, reflecting the comprehensive economic performance of the region [28]. For a long time, the central government has been evaluating the performance of local governments based on such factors as GDP growth rate, investment scale, and fiscal revenue, which mainly reflect the economic quantity and growth rate. It is this single assessment system focusing on economic factors that has led local governments to formulate a GDP-oriented development model, while seriously ignoring environmental protection issues [29]. Although this kind of administrative system can maximize economic growth, it has brought serious environmental risks and ultimately led to the “tragedy of the commons”. After all, the benefits from achieving economic growth are shared by the region, but the increase or decrease in greenhouse gas emissions such as carbon dioxide caused by it affect the whole country, and the consequences are shared by all regions. Even if there is a good low-carbon economic policy, this administrative system can not effectively form policy synergy, nor can it fully exert policy effectiveness.

In addition, the Liaoning Provincial Government has long taken the pursuit of short-term economic benefits as its development goal. Low-carbon economy means strongly supporting enterprises’ innovation and research and development of energy-saving and emission reduction technologies, new energy technologies, carbon capture and storage, and other low-carbon technologies. During this period, a lot of time and capital costs will be spent. In other words, a low-carbon economy is mainly a long-term benefit, which is difficult to achieve in the short term. This has led to insufficient policy support from the Liaoning Provincial Government for the low-carbon economy, and it is difficult to effectively carry out energy conservation, emission reduction, and environmental governance. In this regard, the administrative system must be reasonably positioned around the direction, task, and effectiveness of low-carbon economic policies, which are closely related to society, the market, citizens, and other subjects. If there is no administrative system in line with the low-carbon economic policy, the administrative staff cannot exercise their powers, the policy cannot play its role, and the administrative system will lose its significance. Therefore, the establishment, reform, and improvement of the administrative system are always accompanied by the design, implementation, and optimization of low-carbon economic policies.

### 3.4. The Low-Carbon Technology

Low-carbon technology innovation is fundamental to the development of the low-carbon economy. The low-carbon technologies mainly include building energy-saving technologies, carbon capture and storage technologies, new energy technologies, and emission reduction technologies. There has been a lack of advanced technologies for low-carbon emission reduction in Liaoning Province. This mainly stems from the following factors: first, the Liaoning Provincial Government mainly purchases and introduces low-carbon technologies through commercial means, but the funds for this are insufficient. Second, the financial support for independent research and development of low-carbon technologies is obviously insufficient also. Third, there are not enough scientific researchers to develop low-carbon technologies. Currently, the Liaoning Provincial Government’s salary for researchers is too low, and the protection of intellectual property rights is not enough, which makes researchers lack sufficient motivation for low-carbon technology research and development [30].

### 3.5. The Low-Carbon Concept

Corporations mostly ignore social responsibility. It is the enterprise, the important subject of social production activities, which consumes a large amount of carbon in the production of products and provision of services, which in turn leads to increased emissions of carbon dioxide and causes global climate change. However, at present, most enterprises in Liaoning Province, especially small and medium-sized enterprises, do not put effort into the low-carbon goal of corporate production. This situation is mainly attributed to the fact that companies do not pay attention to their social responsibility. In essence, linking CSR to low-carbon production and sales will not only help achieve low-carbon goals, but also obtain improved economic performance, stakeholder support, and social reputation [31].

Most of the public lack low-carbon awareness. Nowadays, energy-consuming products such as large-displacement cars and large buildings have become the common consumption mode pursued by the public, but they have not realized that this is a high-carbon lifestyle with high energy consumption and high cost. The construction of low-carbon cities and low-carbon provinces is not achieved overnight. It needs to form a series of low-carbon economic regions on the basis of changing the concept of economic growth and improving the awareness of low-carbon life, and then take these regions as the center to drive the development of the low-carbon economy of the whole city and the whole province, so as to finally achieve the goal of building an ecological province. In order to take the lead in building a low-carbon economic province, the relatively mature low-carbon economic region in Liaoning Province is the software industrial belt of Lvshun South Road, Dalian High-tech Zone, and the “five points and one line” Liaoning coastal low-carbon economic belt included in the national development strategy in 2010 [32]. In 2014, Tencent Computer Systems Co., Ltd. (Shenzhen, China) released a low-carbon knowledge questionnaire. Of the 1856 people who participated in the questionnaire, only 22.92% said that they clearly know what a low-carbon lifestyle is, 55.06% said they do not fully understand, and the remaining 22.02% said they do not know at all [33]. This shows that the general population of China has no clear understanding of the concept of low carbon. On the contrary, the high-carbon behavior of the public in Liaoning Province is not common in daily life, such as disposable tableware in restaurants and paper waste in offices. The public does not even know the consequences of the high-carbon consumption caused by the above behaviors. Although the Liaoning Provincial Government even requires limited or compulsory procurement of energy-saving products and regular policy publicity and training, the public’s awareness of low-carbon environmental protection in Liaoning Province and even the whole country still needs to be improved [34]

**Figure 2:**
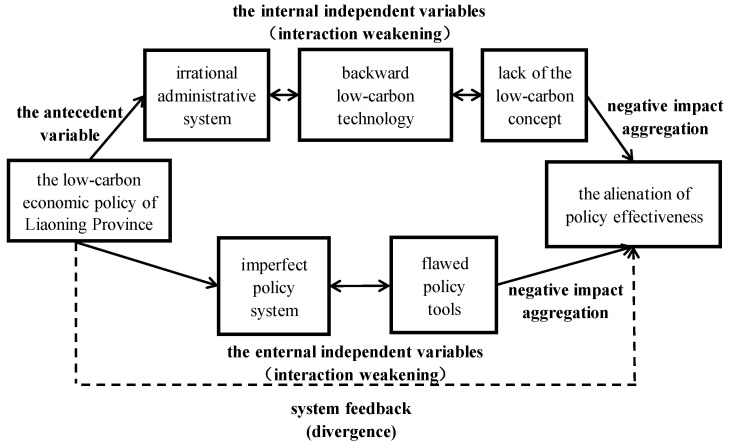
The multi-factor confrontation of policy effectiveness in a low-carbon economy.

Overall, the linkage of the policy system, the administrative system, policy tools, low-carbon technologies, and the low-carbon concept will jointly affect the policy effectiveness of the low-carbon economy in Liaoning Province. When these factors are inhibited and lead to the aggregation of negative effects, a state of confrontation between internal factors and external factors is formed. For further detail on the multi-factor confrontation of policy effectiveness in a low-carbon economy, we refer to Figure 2.

**Figure 3:**
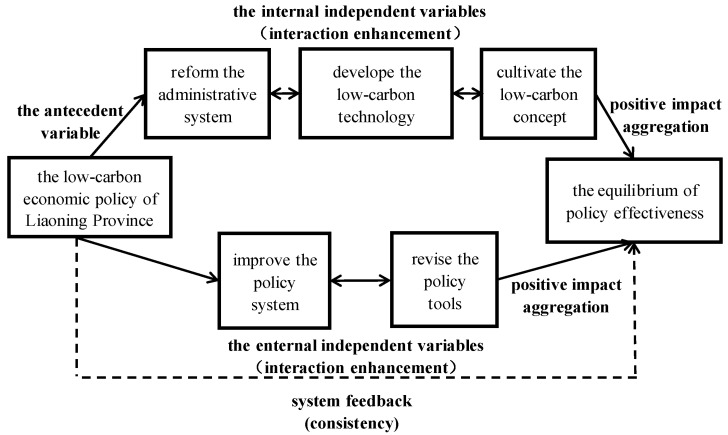
The ideal state of multi-factor linkage of policy effectiveness in a low-carbon economy.

The path to balance the policy effectiveness of the low-carbon economy in Liaoning Province should be to revise the policy system, the administrative system, the policy tools, the low-carbon technologies, and the low-carbon concept, respectively, so that the confrontation between internal and external factors in the multi-factor linkage mechanism is weakened, thus maximizing the effectiveness of the limited combined resources. For further detail on the ideal state of multi-factor linkage of policy effectiveness in a low-carbon economy, we refer to Figure 3.

## 4. The Mathematical Model

The inhibition and equilibrium of low-carbon economic policy effectiveness involve a combination of the policy system, policy tools, the administrative system, low-carbon technology, and the low-carbon concept. In the final analysis, it is a problem in the field of economics. The purpose of balancing the effectiveness of low-carbon economic policies is to meet as many conflicting interests as possible with minimum sacrifice and waste. Therefore, on the basis of the previous discussion, in order to obtain a more reasonable answer, this study must use mathematical models to further consider the process of inhibition and achieving an equilibrium the effectiveness of low-carbon economic policies. This study assumes the context of the low-carbon economic policy of Liaoning Province, selects the administrative system (*R*_1_), the low-carbon technology (*R*_2_), and the low-carbon concept (*R*_3_) as the same set of independent variables, selects the policy system (*R*_4_), and policy tools (*R*_5_) as another set of independent variables, and selects the equilibrium degree of the policy effectiveness of the low-carbon economy in Liaoning Province (*R*) as the dependent variable. This mathematical model maximizes the objective function by showing the game and derivation process of each factor. Specifically, the 3 variables *R_i_* (*i* = 1, 2, 3) can be expressed as:

(1) $$R_{i}=\mu_{i}+\alpha_{i1}E_{1}+\alpha_{i2}E_{2}+\alpha_{i3}E_{3}+\varepsilon_{i}$$

They are expanded as follows:

(2) $$\left[\begin{array}{l} R_{1}\\ R_{2}\\ R_{3} \end{array}\right]=\left[\begin{array}{l} \mu_{1}\\ \mu_{2}\\ \mu_{3} \end{array}\right]+\left[\begin{array}{l} \alpha_{11}\alpha_{12}\alpha_{13}\\ \alpha_{21}\alpha_{22}\alpha_{23}\\ \alpha_{31}\alpha_{32}\alpha_{33} \end{array}\right]\left[\begin{array}{l} E_{1}\\ E_{2}\\ E_{3} \end{array}\right]+\left[\begin{array}{l} \varepsilon_{1}\\ \varepsilon_{2}\\ \varepsilon_{3} \end{array}\right]$$

Similarly, the 2 variables (*R_i_* = 4, 5) can be expressed as:

(3) $$R_{i}=\mu_{i}+\alpha_{i1}E_{1}+\alpha_{i2}E_{2}+\varepsilon_{i}$$

They are expanded as follows:

(4) $$\left[\begin{array}{l} R_{4}\\ R_{5} \end{array}\right]=\left[\begin{array}{l} \mu_{4}\\ \mu_{5} \end{array}\right]+\left[\begin{array}{l} \alpha_{41}\alpha_{42}\\ \alpha_{51}\alpha_{52} \end{array}\right]\left[\begin{array}{l} E_{4}\\ E_{5} \end{array}\right]+\left[\begin{array}{l} \varepsilon_{4}\\ \varepsilon_{5} \end{array}\right]$$

Combining the above equations, they can be further simplified as:

(5) R−μ=ΛE+ε

After the above derivation, we can solve the efficiency balance of the multi-factor model, adjust the combination of the policy system, the policy tools, the administrative system, the low-carbon technology, and the low-carbon concept into a reasonable interaction relationship, measure the impact of different factor combinations on the efficiency of Liaoning’s low-carbon economic policy, and grasp the evaluation function and operation logic of the multi-factor linkage mechanism. The correlation between these external variables and internal variables is not only affected by the force of the mechanism generated by the overall variable combination but also may be directly or indirectly affected by other factors in the mechanism. For example, the choice of policy tools is easily affected by the low-carbon concept, and different concepts lead to different preferences for tax policies, fiscal policies, etc. The greater the correlation of variables among each other, the greater the influence of the policy portfolio showing a positive correlation and cross-cutting trend. At this time, the policy combination can be used as the best plan to balance the effectiveness of low-carbon economic policies in Liaoning Province. This multi-factor linkage mechanism aims to improve the policy factors of each project, form an interactive and balanced whole, and gradually transform some inhibited efficiency into a relatively balanced or absolutely balanced state. From the perspective of the design and implementation of low-carbon economic policies in Liaoning Province, in recent years, the Liaoning Provincial Government has regularly verified and objectively evaluated the effects of various low-carbon economic policies, adjusted the policy mix and policy direction in time, so as to release the optimization of policy effectiveness. These practices have proved that the multi-factor linkage mechanism is feasible for balancing the efficiency of a low-carbon economy and is of great help to solve the development of the low-carbon economy.

However, the equilibrium of this multi-factor linkage mechanism also presents a relative and phased result. Even if equilibrium is achieved, it may be transitory and become unbalanced under the influence of changing external conditions. This involves the issue of economic externality benefits, which may lead to the inhibition of the efficiency of low-carbon economic policies in Liaoning Province, thus causing externality problems. Liaoning Provincial Government may also encounter market failure, rent-seeking behavior, opportunity cost, and other problems in the process of launching low-carbon economic policies, which will lead to direct or indirect multilateral effects between internal and external variables. Therefore, based on the previous mathematical model, we also assume the context of the low-carbon economic policy of Liaoning Province, set the objective of the function as Y, the external benefit as EXE, the internal benefit as INE, and set INE = I (X), then the efficiency function of the low-carbon economic policy of Liaoning Province can be interpreted as:

Y(X) = INE + EXE(6)

Then, let ε = EXE/INE, where ε is the externality coefficient, which represents the relative degree of external effects when a particular program is running. Thus the internalities and the externalities can be related through the externality coefficient ε. If the internal benefits of the subject A in a particular program is Y, then the external benefits function can be written as:

EXE = ε (X) × Y(7)

Objectively, determining the externality coefficient ε may be more valuable than measuring external interests, because in most cases, the internal interests of a specific project operation are clear, but the external interests are often difficult to determine and unpredictable. However, with this externality coefficient ε, we can analyze the positive and negative qualitative and strong and weak quantitative conditions of the externalities in the multi-factor linkage mechanism, so that the policy portfolio can obtain more solutions when faced with economic externalities. Therefore, for the policy effectiveness of the low-carbon economy in Liaoning Province, it is more valuable to determine the externality coefficient than to measure the internal income. For the policies of Liaoning Province, the low-carbon economic policy is the inevitable choice for the development of the public economy, which not only needs to meet the requirements of the market economy but also needs to highlight social justice. This determines that low-carbon economic policies have strong economic externalities. If too much consideration is given to private interests, public responsibilities will be reduced, and policy effectiveness will be more inhibited. Thus, this multi-factor linkage mechanism should not only promote the optimal allocation of various factors to form a balanced policy effectiveness, but also accelerate the elimination of some factors and constantly resolve the problem of economic externalities.

Specifically, we can analyze whether the impact of externalities is positive or negative according to the value of ε.

When ε > 0, it means EXE > 0, which indicates positive externality, implying a positive impact.

When ε < 0, it means EXE < 0, which indicates negative externality, implying a negative impact

When ε = 0, it means EXE = 0, which indicates no externality, implying no impact.

Furthermore, whether the effect of externality is strong or weak can be deduced according to the absolute value of ε.

When |ε| > 1, it means that the externality effect is large, implying a strong externality.

When |ε| < 1, it means that the externality effect is small, implying a weak externality.

When |ε| = 1, it means that the effect of externality is comparable to the internal gain, implying a relatively offsetting effect.

In summary, we can assess the specific situation of the externality impact of the multi-factor linkage mechanism through the externality coefficient ε, so as to provide more solution paths for Liaoning Provincial Government.

## 5. Recommendation

We put forward the following policy recommendations in order to promote the equilibrium of policy effectiveness of the low-carbon economy in Liaoning Province.

### 5.1. Improving the Policy System

The Liaoning Provincial Government should formulate a special general policy for the development of a low-carbon economy. In 2009, the state promulgated the “Law of the People’s Republic of China on the Promotion of Circular Economy”, which provides a guideline for the development of the low-carbon economy. Based on this, Liaoning Provincial Government should make reference to this law and formulate a special regulation according to the current situation of energy consumption structure and low-carbon economy development of Liaoning Province [35], such as the Law on the Promotion of Low-carbon Economy in Liaoning Province, so as to provide general guidance for the series of policies formulated for the development of the low-carbon economy in Liaoning Province. In addition, with the guidance of this general policy, the existing policies of Liaoning Province should be optimized and amended so that they can cooperate and complement each other effectively and also do not conflict with the general policy.

The Liaoning Provincial Government should refine the policy rules based on the actual situation of low-carbon economic development in Liaoning Province. Taking industrial policy as an example, at the UN Climate Ambition Summit in 2020, China proposed that the total installed capacity of wind and solar power will reach more than 1.2 billion kilowatts in 2030 [36], which means that the installed capacity of wind and solar power in China should be tripled in the next 10 years. This is actually an opportunity for the development of a low-carbon economy in Liaoning Province because Liaoning Province has sufficient wind and solar energy resources. For example, in 2019, the installed capacity of wind energy was 9.8 million kilowatts [37], which reflects that wind energy in Liaoning Province has a large potential for development. Therefore, the Liaoning Provincial Government should undertake planning and refinement of industrial policies in advance, so that the industrial policies of Liaoning Province can focus on promoting new energy industries such as wind energy, solar energy, and nuclear energy, thus creating a new engine of low-carbon economic growth in Liaoning Province.

### 5.2. Optimizing the Administrative System

Changing the local government’s performance concept of pursuing GDP is the core of enhancing the policy effectiveness of the low-carbon economy in Liaoning Province. In response, we propose the following policy recommendations. First, the assessment of the local government’s performance should consider not only economic factors but also environmental protection factors such as resource utilization, energy conservation, and emission reduction [38], so as to motivate local governments to vigorously develop the low-carbon economy. Second, each local government should apply different levels of performance evaluation criteria according to the actual situation. For example, Liaoning Province consumes more energy than other provinces, so the evaluation criteria for Liaoning government’s performance in developing the low-carbon economy should be stricter. Moreover, each province has its own different emission reduction tasks, and the central government should give policy preferences, such as tax breaks, to those provinces that exceed their emission reduction tasks, while the central government should be accountable for those provinces that fail to complete their emission reduction tasks. Third, the education and training of Liaoning Provincial Government staff should be strengthened so that they can correctly understand the substance and spirit of the low-carbon economic policy, and then formulate and implement the low-carbon economic policy in a rational manner.

### 5.3. Perfecting the Policy Tools

Regarding fiscal tools, firstly, the state should increase investment support for low-carbon economic development in Liaoning Province. Compared with other provinces, the task of low-carbon economic development in Liaoning Province is more severe and requires more financial support. Therefore, the state should increase financial support to Liaoning Province to promote industrial structure optimization and energy structure transformation. Secondly, in addition to direct support for new energy products, the state and Liaoning Provincial Government should also increase investment support for supporting facilities for new energy products. For example, Liaoning Provincial Government has introduced a series of policies to encourage people to buy new energy vehicles by reducing vehicle purchase tax, but Liaoning Province has not yet established charging facilities and power grids in a timely manner [39]. Overall, strong financial support is necessary to ensure that new energy products can be promoted and used smoothly.

Regarding taxation tools, firstly, the preferential taxation policy should be improved. The national government and Liaoning Provincial Government should clearly define what kind of “scientific research activities” are eligible for tax incentives. We suggest that the policy norms define “scientific research activities” in the form of both general and enumerated provisions. Additionally, Liaoning Provincial Government should simplify the audit procedure for scientific research enterprises to apply for tax incentives, so as to stimulate the enthusiasm of the enterprises for energy saving and technological innovation. Secondly, the punitive tax policy should be optimized. On the one hand, the scope of some taxes should be expanded, and the tax rate should be moderately increased. Specifically, coal consumption should be included in the scope of consumption tax, and the minimum tax rate of coal resource tax should be increased from 2% to 6%, which is at least the same as the minimum tax rate of 6% for oil and natural gas. Furthermore, high-carbon products should be taxed at all stages, including the development stage, the production and processing stage, the sales stage, and the recycling stage, so as to limit the development of a high-carbon economy to the greatest extent. On the other hand, a carbon tax should be set up [40]. A carbon tax is a tax specifically for carbon emissions, which can promote the development of the clean energy industry. China should consider Liaoning Province as the first pilot for levying carbon tax and set a reasonable levy scope and tax rate in accordance with the situation of Liaoning Province so that the Liaoning Provincial Government can accumulate experience in levying carbon tax and low-carbon economic development.

Regarding financial instruments, first, the government should take measures to change the current situation where financial institutions are reluctant to lend to low-carbon enterprises. For instance, the government could provide guarantees actively for the loans of enterprises producing new energy-saving products to reduce the loan risk of financial institutions, thus enhancing the motivation of financial institutions to lend to low-carbon enterprises. In addition, the government can build a green evaluation and reward system, meaning that financial institutions that provide loans actively to low-carbon enterprises can be given preferential tax support and other rewards [41]. Second, the government should establish a tracking and supervision mechanism for financial institutions, requiring enterprises to regularly disclose information to financial institutions after borrowing from them, such as how is the enterprise operating and where is the loan going, so as to prevent enterprises from fraudulent loans under the pretext of developing the low-carbon economy. Third, the state should make greater efforts to support the development of the financial industry in Liaoning Province, such as building diversified financial institutions and selling diversified financial products, so as to increase the borrowing channels of low-carbon enterprises.

### 5.4. Strengthening the Research of the Low-Carbon Technologies

Firstly, the government should increase financial support for the introduction of low-carbon technologies, and also prioritize the technologies that are most beneficial to the development of the low-carbon economy in Liaoning Province. For example, at present, the development of clean energy technology and energy-saving technology in Liaoning Province is still lagging behind [42], so the Liaoning Provincial Government should give priority to introducing these two kinds of technologies. Secondly, more research funds and advanced research equipment should be provided by the government to create a better research environment. Thirdly, the government should take active measures to attract outstanding researchers to research and develop low-carbon technologies. For instance, the government could improve the salary of the researchers and protect the intellectual property rights of the researchers as much as possible. Moreover, the Liaoning Provincial Government should actively cooperate with the research institutions and universities in Liaoning Province, such as Dalian Institute of Chemical Technology, Institute of Ecology of Chinese Academy of Sciences, Northeastern University, and Dalian University of Technology, to jointly develop low-carbon technologies.

### 5.5. Cultivating the Concept of the Low-Carbon Economy

In order to inspire enterprises to fulfill their social responsibilities actively and individuals to establish low-carbon economy awareness, it is necessary to cultivate the concept of the low-carbon economy through the following ways. First, the government could propagandize the concept of a low-carbon economy actively through the media, such as television, radio, and WeChat [43]. Second, education on the concept of the low-carbon economy should be strengthened by the government. For example, the government can require that the knowledge related to energy conservation and emission reduction be written into textbooks and that schools set up environmental protection courses. Moreover, the government can require schools and other social organizations to educate the public about energy conservation and carbon reduction by holding lectures and competitions regularly, so that the concept of the low-carbon economy penetrates into people’s daily lives. Third, the government can establish some special holidays, such as the “one hour of electricity saving holiday”, which calls on the public to turn off air conditioners, TVs, fans, and other household appliances for one hour, so as to cultivate good habits of energy saving and emission reduction.

## 6. Conclusions

The inhibition of the policy effectiveness of the low-carbon economy in Liaoning Province of China is also a difficulty and challenge that other developing countries are likely to encounter. Taking Liaoning Province as an example, this study reviews the phenomenon of the inhibition of policy effectiveness of the low-carbon economy in Liaoning Province and discusses the problems of the policy systems, the administrative systems, the policy tools, the low-carbon technologies, and the low-carbon concept that lead to the inhibition of policy effectiveness. The multi-factor linkage models and a special mathematical model that maximize the equilibrium of policy effectiveness are established by applying the modified Neoclassical Realist Theory and the economic method, and the strategies to refine and optimize the policy systems, the administrative systems, the policy tools, the low-carbon technologies, and the low-carbon concept are proposed. Similarly to China, some developing countries also face the dilemma of inhibiting the policy effectiveness of the low-carbon economy, such as India, Iran, and Mexico. Therefore, this study provides good enlightenment for these developing countries to maximize the equilibrium of policy effectiveness of the low-carbon economy, mitigate the high-carbon emission problem, and develop the low-carbon economy.

The strategies and technological approaches proposed in this paper may be far from sufficient to change global warming and repair environmental damage, but they are significant for achieving sustainable socioeconomic development, moving toward ecological civilization, and safeguarding public health. Given that the development of the low-carbon economy to promote public health is a protracted battle, the government has a long way to go to make and revise policies continuously for the low-carbon economy.

## Data Availability

Not applicable.
